# RUNX Family Participates in the Regulation of p53-Dependent DNA Damage Response

**DOI:** 10.1155/2013/271347

**Published:** 2013-09-03

**Authors:** Toshinori Ozaki, Akira Nakagawara, Hiroki Nagase

**Affiliations:** ^1^Laboratory of DNA Damage Signaling, Chiba Cancer Center Research Institute, 666-2 Nitona, Chuohku, Chiba 260-8717, Japan; ^2^Laboratory of Innovative Cancer Therapeutics, Chiba Cancer Center Research Institute, Chiba 260-8717, Japan; ^3^Laboratory of Cancer Genetics, Chiba Cancer Center Research Institute, Chiba 260-8717, Japan

## Abstract

A proper DNA damage response (DDR), which monitors and maintains the genomic integrity, has been considered to be a critical barrier against genetic alterations to prevent tumor initiation and progression. The representative tumor suppressor p53 plays an important role in the regulation of DNA damage response. When cells receive DNA damage, p53 is quickly activated and induces cell cycle arrest and/or apoptotic cell death through transactivating its target genes implicated in the promotion of cell cycle arrest and/or apoptotic cell death such as *p21^WAF1^*, *BAX*, and *PUMA*. Accumulating evidence strongly suggests that DNA damage-mediated activation as well as induction of p53 is regulated by posttranslational modifications and also by protein-protein interaction. Loss of p53 activity confers growth advantage and ensures survival in cancer cells by inhibiting apoptotic response required for tumor suppression. RUNX family, which is composed of RUNX1, RUNX2, and RUNX3, is a sequence-specific transcription factor and is closely involved in a variety of cellular processes including development, differentiation, and/or tumorigenesis. In this review, we describe a background of p53 and a functional collaboration between p53 and RUNX family in response to DNA damage.

## 1. Introduction

The initial chromatin-associated molecular event upon DNA damage is the activated ataxia telangiectasia mutated- (ATM-) mediated phosphorylation of histone variant H2AX (*γ*H2AX), which marks the sites of DNA damage (nuclear foci) [1]. Then a large nuclear adaptor protein termed mediator of DNA damage response 1 (MDC1)/nuclear factor with BRCT domain 1(NFBD1) associates with the sites of DNA damage and facilitates the recruitment of DNA repair machinery including MRN (MRE11, Rad50, and NBS1) complex onto nuclear foci to repair damaged DNA [2–5]. When cells receive the repairable DNA damage, cell cycle arrest takes place to save time to correctly repair damaged DNA, and then cells with repaired DNA reenter into the normal cell cycle (cell survival). In contrast, when cells receive the severer DNA damage, which cannot be repaired, cells with seriously damaged DNA undergo apoptotic cell death and are then eliminated from tissues (cell death). Thus, the appropriate DNA damage response plays an important role to maintain genomic integrity to avoid genomic aberrations such as deletions and mutations, which result in genomic instability and finally induce tumor formation [6–8].

p53 has been initially identified as a 53 KDa of nuclear protein which tightly associated with oncogenic simian virus 40 (SV40) large T antigen [9–13]. Structural analysis revealed that p53 is composed of three functional domains including NH_2_-terminal acidic transactivation domain (TA, amino acid residues 1–45), central sequence-specific DNA-binding domain (DB, amino acid residues 102–292), and COOH-terminal oligomerization domain (OD, amino acid residues 319–359) [6–8], suggesting that p53 could act as a nuclear transcription factor. Indeed, p53 had an ability to bind to salmon sperm DNA *in vitro* [14]. Tetramerization, which is mediated by its oligomerization domain, is essential for the ability of p53 to positively regulate gene expression [15]. In addition to these three characteristic domains, p53 contains three nuclear localization signals (NLS, amino acid residues 305–322, 369–375, and 379–384) [16], a Leu-rich nuclear export signal (NES, amino acid residues 339–352) [17], and a Pro-rich domain (amino acid residues 63–97) [18]. Subsequent studies demonstrated that p53 has an oncogenic potential and has a capacity to promote tumor growth [19]. Intriguingly, p53 was easily detectable in a variety of tumor-derived cell lines, implying that p53 is abundantly expressed in these cancerous cells [20]. Based on these observations, initially isolated p53 came to be classified as an oncogene product [21]. However, this classical point of view has been challenged by the findings demonstrating that the initially identified p53 is a mutant form of p53 [22]. A number of studies clearly showed that, in contrast to mutant form of p53, wild-type p53 is able to suppress aberrant cell growth of transformed cells as well as tumors [23, 24]. Of note, *p53*-deficient mice frequently developed spontaneous tumors [25], indicating strongly that wild-type p53 acts as a tumor suppressor. 

Extensive mutation searches revealed that nearly half of human tumors carry *p53* mutations except human neuroblastoma, rhabdomyosarcoma, and melanoma [26]. el-Deiry et al. described that cyclin-dependent protein kinase (CDK) inhibitor termed wild-type p53-activated fragment 1 (p21^WAF1^) is one of p53-inducible gene products, and proposed that p53 recognizes and binds to a consensus sequence motif made of tandem 10 bp elements (RRRCWWGYYY) separated by 1–13 bp found within the promoter regions of p53-target genes [27, 28]. In accordance with their findings, accumulating evidence suggests that p53 is a sequence-specific nuclear transcription factor [29, 30]. To date, numerous p53-inducible gene products have been identified including proapoptotic BAX (Bcl2-associated X protein), NOXA (Latin for damage), PUMA (p53-upregulated modulator of apoptosis) and p53AIP1 (p53-regulated apoptosis-inducing protein 1) [31–34] and gene products inducing cell cycle arrest such as p21^WAF1^ and 14-3-3*σ* [35]. Importantly, 95% of *p53* mutations have been detected within the genomic region encoding its central sequence-specific DNA-binding domain [26]. Among them, 20% of mutations accumulate within six hot-spots (amino acid residues 175, 245, 248, 249, 273, and 282), which are most targeted in *p53* gene. These mutations disrupt the native conformation of the sequence-specific DNA-binding domain of wild-type p53 and result in the loss of its sequence-specific DNA-binding activity [30]. Several lines of evidence indicated that the sequence-specific DNA-binding ability of p53 is tightly linked to its proapoptotic activity [30], suggesting that blocking p53-dependent sequence-specific transcription is a critical event in tumorigenesis. Mutant forms of p53 lose the activity to prevent uncontrolled cell growth and protect cells from genomic alterations [36]. Thus, mutant forms of p53 lack its critical function to maintain genomic integrity in response to DNA damage. 

Human runt-related transcription factor (RUNX) family is composed of three members including RUNX1, RUNX2, and RUNX3. RUNX family is highly conserved in their runt homology domain, which is involved in the sequence-specific DNA binding and heterodimerization with the common co-factor CBF*β* [37]. In addition to runt domain, RUNX family also possesses the other functional subdomains such as a large transactivation domain in COOH-terminal part and an inhibitory domain at COOH-terminal end of the transactivation domain [37]. A growing body of evidence demonstrated that each of RUNX family members has a distinct biological function. For example, RUNX1 is required for the establishment of the hematopoietic stem cells and is a frequent target of chromosomal gene translocations in hematopoietic malignancies [38–40]. On the other hand, RUNX2 plays an essential role in the promotion of both osteoblast and terminal chondrocyte differentiation and is also responsible for bone formation and mineralization *in vivo* [41, 42]. Since *RUNX3*-deficient mice displayed hyperplasia in gastric mucosa caused by reduced apoptosis and the stimulated growth of the gastric epithelial cells, RUNX3 has been considered to be a tumor suppressor for human gastric cancer [43]. Although the functional contribution of RUNX family members to the regulation of DNA damage response has been elusive, we have found for the first time that RUNX1 as well as RUNX3 acts as a coactivator for p53 in response to DNA damage, whereas RUNX2 represses p53-dependent apoptotic cell death following DNA damage. Based on our recent findings, RUNX family might participate in the regulation of p53-mediated DNA damage response.

## 2. Induction of p53 in Response to DNA Damage

Since p53 plays a critical role in growth suppression and disruption of balanced p53 activity as well as amount would lead to tumor development, under normal physiological conditions, p53 is kept at extremely low level and thus barely detectable. Upon multiple cellular stresses including DNA damage, oncogene activation, hypoxia, nucleotide imbalance and oxidative damage, p53 is rapidly induced to largely accumulate in cell nucleus through sequential post-translational modifications such as phosphorylation (Ser-15, Ser-20, and Ser-46) and acetylation (Lys-370, Lys-372, Lys-373, Lys-381, and Lys-392) [6–8]. These chemical modifications convert p53 from latent form to active one *via* the dynamic conformation shift. DNA damage-mediated induction of p53 is mainly regulated at protein level but not at transcription level. RING-finger type E3 ubiquitin protein ligase Murine double minute 2 (MDM2), which has an oncogenic potential, binds to NH_2_-terminal portion of p53, catalyses ubiquitination of six Lys residues (Lys-370, Lys-372, Lys-373, Lys-381, Lys-382, and Lys-386) at its COOH-terminal region, and then promotes its proteolytic degradation through proteasome [44–46]. Since MDM2 is one of p53-induced gene products, MDM2 participates in a negative autoregulatory feedback loop, which controls p53 expression level [47]. It is likely that MDM2 monitors the amount as well as the activity of p53 in response to DNA damage, thereby prohibiting inappropriate apoptotic cell death by overactive p53. As NH_2_-terminal phosphorylation of p53 facilitates the dissociation of MDM2 from p53 and also COOH-terminal acetylation inhibits MDM-mediated p53 ubiquitination following DNA damage, p53 becomes stable in response to DNA damage. Recently, Meek and Hupp described that, in response to DNA damage, ATM and CHK (checkpoint kinase) phosphorylate multiple Ser residues within or close to RING-finger domain of MDM2 thereby destabilizing MDM2 [48]. Thus, the intracellular amount of MDM2 available appears to be critical in determining the expression level of p53. Additionally, MDM2 masks the important amino acid residues of NH_2_-terminal transactivation domain of p53, and therefore the sequence-specific transactivation ability of p53 is significantly attenuated by MDM2 [49].

## 3. Cell Survival or Cell Death following DNA Damage

Biological consequences in response to DNA damage might be dependent on the degree and/or the nature of DNA damage. When DNA damage is severe and repair is impossible, activated p53 exerts its strong proapoptotic function to remove cells with seriously damaged DNA through apoptotic cell death. Under these conditions, p53 transactivates pro-apoptotic key target genes including *BAX*, *PUMA*, *NOXA*, and *p53AIP1* [6–8]. Among them, PUMA is a direct activator of BAX [50]. The collaboration of these pro-apoptotic gene products contributes to the disruption of mitochondrial membrane potential, thereby releasing cytochrome c from mitochondrial intermembrane space. Then the release of cytochrome c causes apoptotic protease activating factor 1 (Apaf1) oligomerization, resulting in apoptosome formation. This complex, in turn, recruits and activates procaspase-9, which then activates executioner caspases-3 and caspase-7, which is a critical step in p53-dependent pro-apoptotic pathway [51, 52].

**Figure 1:**
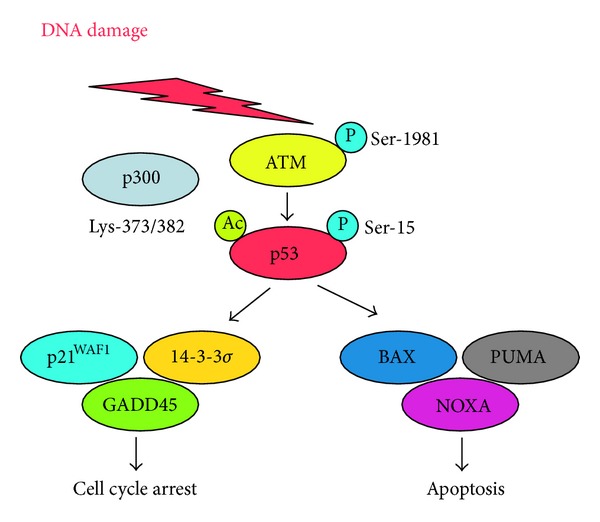
p53-dependent DNA damage response. Upon DNA damage, p53 is phosphorylated at Ser-15 and acetylated at Lys-373/382 by ATM and p300, respectively. Activated form of p53 induces cell cycle arrest and/or apoptotic cell death.

In contrast, when cells receive the repairable DNA damage, p53 induces G1/S and/or G2/M cell cycle arrest through the transactivation of *p21* ^*WAF1*^, *p53R2 *(p53-inducible ribonucleotide reductase small subunit), *14-3-3*σ** and *GADD45* (Growth arrest and DNA damage 45) implicated in the promotion of cell cycle arrest and DNA repair [53, 54]. After DNA repair is completed, cells reenter into normal cell cycle to faithfully transmit genetic information to their daughter cells. During this repair process, mispaired DNA bases are replaced with correct bases by mismatch repair (MMR), and small chemical alterations of DNA bases are repaired by base excision repair (BER) through excision of the damaged base [55, 56]. We have previously found that MDC1/NFBD1 attenuates ATM-dependent phosphorylation of p53 at Ser-15 during the early phase of DNA damage response, suggesting that MDC1/NFBD has an ability to inhibit pro-apoptotic activity of p53 and save time to repair damaged DNA [57]. At the late phase of DNA damage response, the expression level of MDC1/NFBD1 was reduced, p53 dissociated from MDC1/NFBD1, and p53 exerted its pro-apoptotic activity. *MDC1*/*NFBD1*-deficient mice exhibited chromosome instability and DNA repair defects. In addition, it has been shown that p53 transrepresses some genes such as anti-apoptotic *Bcl-2* [58, 59], indicating that the concerted action of a whole set of p53-induced genes as well as p53-repressed genes triggers a specific biological consequence following DNA damage. Therefore, it is likely that p53 stands at the crossroad between cell survival and cell death in response to DNA damage (Figure 1). However, the molecular basis for the choice between cell cycle arrest and apoptotic cell death induction by p53 is not well understood.

## 4. Protein-Protein Interaction

In addition to the posttranslational modifications, transcriptional as well as pro-apoptotic activity of p53 is also regulated positively or negatively by protein-protein interaction. Certain cellular proteins affect the modification status of p53 following DNA damage. The early study demonstrated that nuclear nonreceptor tyrosine kinase c-Abl negatively regulates cell growth [60]. Subsequent analysis showed that c-Abl, associates with p53 and enhances its sequence-specific transcriptional activation and mutant c-Abl which no longer binds to p53, fails to increase the transcriptional activity of p53 [61]. Additionally, Yuan et al. revealed that c-Abl is required for p53-dependent apoptotic cell death in response to DNA damage [62]. Alternatively, Samuels-Lev et al. found that apoptosis stimulating protein of p53 (ASPP), which contains ankyrin repeats, SH3 domain and Pro-rich domains, interacts with p53 and enhances its sequence-specific transactivation as well as its proapoptotic activity [63]. According to their observations, ASPP stimulated p53-dependent apoptotic cell death in response to DNA damage. Recently, Gillotin and Lu described that ASPP forms a complex with p300 histone acetyltransferase and selectively regulates the transcriptional activity of p53 [64]. Yang et al. reported that 14-3-3*σ* is a direct transcriptional target of p53 and interacts with p53 to increase its protein stability through blocking MDM2-mediated ubiquitination of p53 [35]. Consistent with these observations, 14-3-3*σ* has been shown to enhance the transcriptional activity of p53. Since 14-3-3*σ* is one of p53-responsive gene products, 14-3-3*σ* creates a positive auto-regulatory loop in which p53 transactivates *14-3-3*σ** and in turn 14-3-3*σ* enhances p53 activity. Recently, Kim et al. demonstrated that tumor suppressor wilms tumor gene on X chromosome (WTX) associates with DNA-binding domain of p53 and elevates its CBP (CREB-binding protein)/p300-mediated acetylation level at Lys-373/382 [65]. Based on their results, WTX was able to increase the transcriptional and pro-apoptotic activities of p53 through the regulation of the interaction between p53 and CBP [65, 66]. 

Protein-protein interaction does not always enhance p53 activity. As mentioned above, MDM2 binds to NH_2_-terminal transactivation domain of p53 and strongly blocks its transcriptional as well as pro-apoptotic ability [49]. We have previously described that oncogenic polo-like kinase 1 (Plk1) whose expression level is significantly higher in various human tumor tissues as compared with their corresponding normal ones, associates with p53 and inhibits its activity through phosphorylation [67]. Alternatively, it has been shown that silent mating type information regulation 2 homolog 1 (SIRT1) interacts with p53 and inhibits p53 through deacetylation of p53 [68]. Consistent with these observations, the catalytically impaired SIRT1 increased p53-target gene expression after DNA damage [69]. Recently, Jang et al. described that SIRT1 promotes the human liver cancer survival [70] and Cha et al. reported that the expression level of *SIRT1* is a poor prognostic indicator for human gastric carcinomas [71]. 

In general, subcellular localization of p53 might be one of critical determinants for p53 activity. For example, it has been well known that human neuroblastomas express nonfunctional p53 without mutations, which might be due to its abnormal cytoplasmic retention [72]. Intriguingly, Nikolaev et al. discovered a large cytoplasmic protein termed p53-associated, Parlin-like cytoplasmic protein (Parc), which associated with cytoplasmic p53 in neuroblastoma cells [73, 74]. According to their findings, Parc bound to the majority of cytoplasmic p53 and acted as a cytoplasmic anchor protein for p53. In a good agreement with this notion, silencing of Parc induced nuclear access of p53 and promoted apoptotic cell death in neuroblastoma cells. However, knockdown of Parc in hepatocellular cells had an undetectable effect on cytoplasmic p53 [75], suggesting that Parc could act as a cytoplasmic anchor protein for p53 in a neuroblastoma-specific manner. In addition to Parc, SIRT1 has been shown to block nuclear translocation of cytoplasmic p53 in response to the endogenous reactive oxygen species (ROS) [76]. 

## 5. Mutant Form of p53

**Figure 2:**
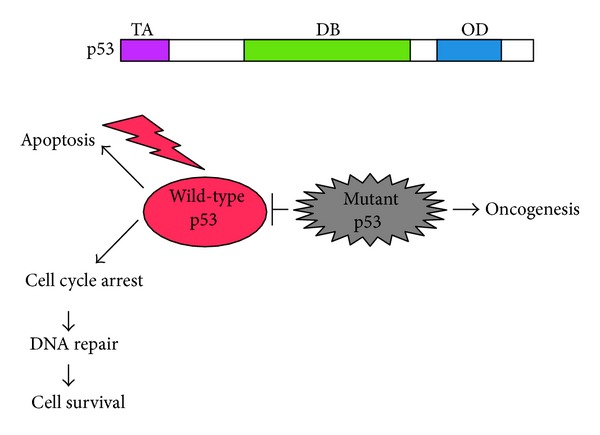
Structure of p53 and dominant-negative behavior of mutant p53 toward wild-type p53. p53 is composed of three functional domains including NH_2_-terminal transactivation domain (TA) followed by central sequence-specific DNA-binding domain (DB) and COOH-terminal oligomerization domain (OD) (upper panel). Lower panel shows the dominant-negative behavior of mutant p53 against wild-type p53.

As mentioned above, *p53* is frequently mutated in human various tumor tissues. Most of *p53* mutations are detectable within the genomic region encoding its sequence-specific DNA-binding domain (exons 5–8), and thus mutant forms of p53 lack the sequence-specific transactivation ability. In a sharp contrast to wild-type p53, mutant forms of p53 display a greatly extended half-life, which might be attributed to the escape from MDM2-mediated ubiquitin/proteasome-dependent proteolytic degradation system [6–8]. Recently, Wiech et al. described that heat shock protein 70 (HSP70) partially inhibits proteasomal degradation of mutant p53 [77]. Furthermore, mutant forms of p53 exhibit a dominant-negative behavior toward wild-type p53 [6–8]. When wild-type p53 and mutant form of p53 are coexpressed in certain cancerous cells, mutant form of p53 strongly prohibits the tumor suppressive activity of wild-type p53 (Figure 2). Indeed, cancerous cells carrying *p53* mutations sometimes display the chemoresistant phenotypes, which might be at least in part due to the presence of mutant p53. Consistent with these observations, many patients with *p53* mutations have an increased resistance to conventional chemotherapy and poorer prognosis than those who have wild-type p53 or no p53 protein [78–81].

In addition to the dominant-negative activity of mutant p53 toward wild-type p53, a growing body of evidence suggests that mutant p53 acquires the alternative gain-of-function activities. Although mutant p53 has been considered to be transcriptionally inactive due to the fact that most p53 mutants fail to transactivate p53-inducible genes, it has been shown that mutant p53 has an ability to regulate the expression of the specific set of genes implicated in tumor initiation and maintenance such as *MDR-1*, c-*Myc*, c-*fos*, and *HSP70* [82–85]. Among them, MDR-1 confers multidrug-resistant phenotype. These findings imply that mutant p53 retains the transactivation potential, whereas its target genes are completely different from those of wild-type p53. Indeed, most promoters activated by mutant p53 lack the sequences similar to the consensus p53-responsive element, suggesting that mutant p53 might regulate transcription through the response elements that are distinct from wild-type p53-target sequence. Of note, these promoters transactivated by various p53 mutants show no sequence homology, indicating that sequence recognition by p53 mutants might be dependent on their specific conformations [86]. 

Nuclear factor Y (NF-Y) is a heterotrimeric transcription factor, which recognizes CCAAT consensus motif, and has been shown to regulate CCAAT-containing promoters including *E2F-1*, cyclin A, *cdc25C*, and *MDR-1* in response to DNA damage [87]. Di Agostino et al. have described that NF-Y associates with mutant p53, and this complex increases the expression of cell cycle-related *cyclin A*, *cyclin B1*, *cdk1*, and *cdc25C* following DNA damage [88]. According to their results, mutant p53/NF-Y complex was recruited onto NF-Y-target promoters together with histone acetyltransferase p300. In contrast, it has been reported that the expression of these cell cycle-related genes is repressed by wild-type p53/NF-Y complex in response to DNA damage [88]. Under their experimental conditions, wild-type p53 formed a complex with NF-Y on CAAT-containing promoters, and this complex recruited histone deacetylase (HDAC) and released histone acetyltransferase. Therefore, it is likely that mutant p53 binds to the similar transcription factors of wild-type p53 and causes the aberrant transcriptional regulation of their target gene expression.

## 6. Possible Therapeutic Strategies

Dysfunction of a proper DNA damage response mediated by wild-type p53 results in the accumulation of various gene mutations and induces genomic instability, thereby promoting tumor formation. As mentioned above, mutant forms of p53 play a critical role in the disruption of p53-dependent appropriate DNA damage response. In contrast to the short half-life of wild-type p53, mutant forms of p53 are extremely stable and have a prolonged half-life. Furthermore, mutant forms of p53 display a dominant-negative behavior toward wild-type p53. Thus, the intracellular balance between mutant p53 and wild-type p53 might be a major determinant of cell fate in response to DNA damage. Since wild-type p53 is quickly degraded by MDM2, extensive efforts have been performed to identify the small compound(s), which could inhibit the interaction between p53 and MDM2. A small molecule termed nutlin, which specifically occupied p53-binding pocket on the surface of MDM2, blocked the interaction between p53 and MDM2 and liberated p53 from MDM2-mediated degradation system, leading to the stabilization, accumulation, and activation of p53 in cancerous cells with wild-type p53 [89, 90]. Nutlin inhibited tumor growth in a nongenotoxic manner in xenografted tumor mice [91]. This nongenotoxic effect of nutlin might be attractive from a therapeutic standpoint. Since the conventional antitumor drugs introduce DNA damage in tumor tissues as well as their surrounding normal ones, it is possible that these treatments might have a potential for secondary malignancies. Surprisingly, Aziz et al. found that, upon repeated exposure of nutlin to certain cancerous cells expressing wild-type p53, a small population of nutlin-resistant cells expands and finally acquires somatic p53 mutations [92]. 

**Figure 3:**
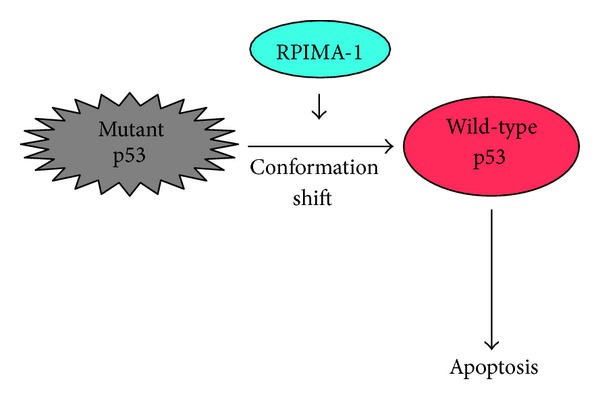
PRIMA-1 reactivates mutant p53. Small chemical compound termed PRIMA-1 has an ability to reactivate mutant p53 through the conformation change.

The previous studies demonstrated that abnormal conformation of certain mutant forms of p53 is reversible under the specific conditions [93], indicating that certain mutant p53 could be reactivated by conformation change. Bykov et al. have extensively screened a library of small compounds in order to find out compound(s) that could restore wild-type function to mutant p53 [94]. According to their results, they finally identified one small compound termed p53 reactivation and induction of massive apoptosis (PRIMA-1), which was able to restore sequence-specific transactivation function and the active conformation to mutant p53 (Figure 3). Furthermore, treatment of PRIMA-1 exhibited a significant anti-tumor effect in mice. Their results raise a possibility that the combination of PRIMA-1 and the conventional chemotherapeutic drugs might be a novel strategy to treat mutant p53-carrying malignant cancers. Bykov et al. found a remarkable synergistic effect *in vitro* between PRIMA-1^Met^ and various antitumor drugs including cisplatin (CDDP), adriamycin (ADR), and camptothecin (CPT) [95]. PRIMA-1^Met^, which was more active than the original compound, is a methylated form of PRIMA-1. Among them, combination of PRIMA^Met^ and cisplatin produced a significantly smaller volume of tumors than those of PRIMA^Met^ or cisplatin treatment alone.

Recently, Wassman et al. utilized a computational method to identify a small molecule(s) which could open p53 core domain flanked by loop L1 and sheet S3 (L1/S3 reactivation pocket) [96]. Finally, they found stictic acid as a p53 reactivation compound. Based on their results, stictic acid enhanced luciferase activity driven by *p21* ^*WAF1*^ promoter in cells expressing R175H mutant of p53. 

## 7. RUNX1 Acts as a Positive Regulator for p53-Dependent Apoptotic Cell Death in Response to DNA Damage

Human runt-related transcription factor (RUNX) family is composed of three members including RUNX1, RUNX2, and RUNX3 [37]. The highly conserved runt domain has been shown to be responsible for both sequence-specific DNA binding and heterodimerization with CBF-*β* [37]. *RUNX1* is the most frequent target for chromosomal translocation in human acute myeloid leukemia (AML), generating the oncogenic fusion proteins such as RUNX1/ETO [38–40]. To date, more than ten RUNX1 fusion proteins have been identified [97]. *RUNX1* has been identified as a gene located at a breakpoint of the chromosomal translocation t(8;21) [38–40]. In addition to the chromosomal translocation, *RUNX1* point mutations are found in sporadic as well as familial myeloid leukemia and also in AML. Subsequent studies revealed that most of *RUNX1* mutations are detectable within the genomic region encoding its runt domain (nearly 80%) [98, 99]. These runt domain mutants lack sequence-specific DNA-binding activity; however, they retain an ability to form a heterodimer with CBF-*β*. RUNX1 mutant binds to CBF-*β* more efficiently than wild-type RUNX1. Since CBF-*β* protects RUNX1 from ubiquitin/proteasome-dependent degradation system, RUNX1 mutant/CBF-*β* heterodimer is much more stable than wild-type RUNX1/CBF-*β* hetero-dimer. Thus, RUNX1 mutant inhibits sequence-specific transcriptional activity of wild-type RUNX1, thereby acting as a dominant-negative inhibitor toward wild-type RUNX1 [100, 101]. Although several lines of evidence strongly suggest that RUNX1 might act as a tumor suppressor, the precise molecular mechanisms of how RUNX1 could exert its tumor-suppressive activity have been elusive. 

Numerous evidences strongly suggest that RUNX1 is required for normal blood development. For example, *RUNX1*-deficient mice displayed a remarkable defect in hematopoiesis [38, 39]. In accordance with these observations, RUNX1 stimulated the transcription of a number of myeloid- and lymphoid-related genes [102, 103]. In addition to the hematopoiesis-specific genes RUNX1 has been shown to be also involved in the regulation of cell cycle-related genes, such as *p21* ^*WAF1*^ [104]. Intriguingly, it has been reported that RUNX1 induces senescence-like growth arrest in primary murine fibroblasts, and this response is lost in cells lacking functional p53 [105, 106]. Recently, Satoh et al. demonstrated that loss of function mutant of RUNX1 attenuates DNA damage-mediated repair response in hematopoietic stem/progenitor cells [107], indicating that RUNX1 might be required for the proper DNA damage response. These results prompted us to investigate whether RUNX1 could participate in p53-dependent apoptotic cell death in response to DNA damage. 

According to our recent results [108], RUNX1 together with p53 accumulated in human osteosarcoma-derived U2OS cell nucleus following ADR exposure, formed a stable complex, and was recruited onto p53-activated promoters including *p21* ^*WAF1*^ and *BAX*, suggesting that RUNX1 might affect the transcriptional activity of p53. Knockdown of RUNX1 resulted in a significant reduction of ADR-mediated apoptotic cell death in association with a remarkable downregulation of p53-responsive gene expression. It was worth noting that RUNX1 associates with p300 and p300-mediated acetylation of p53 at Lys-373/382 following ADR treatment is reduced in RUNX1 knockdown cells. Hence, it is conceivable that RUNX1 acts as a molecular bridge or a scaffolding protein for p53-p300 binding, thereby enabling p300-mediated acetylation of p53 at Lys-373/382 in response to DNA damage. 

## 8. RUNX3 Acts as a Positive Regulator for p53-Dependent Apoptotic Cell Death in Response to DNA Damage

RUNX3 has been shown to be involved in the formation of variety of a human cancers [37]. The initial studies revealed that *RUNX3*-deficient mice develop the hyperplasia in their gastric mucosa, which might be due to the increased proliferation as well as diminished apoptotic cell death [43]. Intriguingly, *RUNX3* is located on human chromosome 1p36, a region which has long been suggested to be a tumor suppressor locus in a variety of human cancers. Although *RUNX3* is infrequently mutated in various human cancers, R122C mutation found within its conserved runt domain, which was identified in a gastric cancer patient, abolished the tumor-suppressive function of RUNX3 in nude mice [43]. The expression level of *RUNX3* in human cancers is kept at extremely low level, and the higher *RUNX3* expression level in human cancers is closely associated with a favorable prognosis with reduced recurrence and better survival rates in patients. On the other hand, lower expression level of *RUNX3* is associated with tumor progression and poor prognosis in various cancers [109–112]. Subsequent studies demonstrated that the combination of hemizygous deletion of *RUNX3* and the hypermethylation of *RUNX3* promoter region contributes to the silencing of *RUNX3* expression in various human cancers [37]. 

 Since RUNX3 is a sequence-specific transcription factor, its nuclear localization is essential for its transcriptional function. In this regard, the mislocalization of RUNX3 might be one of the molecular mechanisms behind the inactivation of RUNX3. Indeed, it has been shown that RUNX3 is frequently detectable in cytoplasm of various human cancers including gastric cancer, colorectal cancer, and breast cancer [113]. Thus, one allele of RUNX3 is silenced through promoter hypermethylation, while the remaining wild-type allele is inactivated through the abnormal cytoplasmic localization of its gene product. 

 RUNX3 activity is highly associated with transforming growth factor *β* (TGF-*β*) signaling, which promotes both cell cycle arrest and apoptotic cell death [114, 115]. Previous studies showed that gastric epithelial cells derived from *RUNX3*-deficient mice are highly resistant to the growth-suppressive and apoptotic cell death-inducing effects of TGF-*β* [43]. Consistent with these observations, Ito et al. found that RUNX3, a downstream of the tumor-suppressive TGF-*β* pathway, antagonizes the oncogenic Wnt pathway in intestinal carcinogenesis, thereby inhibiting the transcriptional activity of *β*-catenin/ T-cell factor 4 (TCF4) [116]. In addition, Yano et al. reported that RUNX3 is required for transcriptional upregulation of proapoptotic BH-3 only protein Bcl-2-interacting mediator of cell death (Bim) during TGF-*β*-induced apoptotic cell death [117]. However, a possible involvement of RUNX3 in the regulation of DNA damage response remains to be unknown.

 Recently, we have found for the first time that RUNX3 knockdown significantly attenuates ADR-mediated apoptotic cell death in *p53*-proficient U2OS and human lung carcinoma A549 cells but not in *p53*-deficient human lung carcinoma H1299 and human osteosarcoma SAOS-2 cells [118]. Of note, knockdown of RUNX3 resulted in a massive reduction of ADR-dependent phosphorylation level of p53 at Ser-15 in association with a remarkable downregulation of various p53-target gene expressions, and forced expression of RUNX3 elevated the ADR-mediated p53 phosphorylation level at Ser-15 in its upstream protein kinase ATM-dependent manner. Under the unstressed conditions, RUNX3 was detected in both cell nucleus and cytoplasm, whereas RUNX3 was induced to translocate into cell nucleus following ADR exposure. Since RUNX3 formed a stable complex with p53 and activated ATM in the presence of ADR, it is likely that RUNX3 assists ADR-mediated phosphorylation of p53 at Ser-15 in response to ADR, indicating that, like RUNX1, RUNX3 acts as a positive regulator for p53 following DNA damage.

## 9. RUNX2 Acts as a Negative Regulator for p53-Dependent Apoptotic Cell Death in Response to DNA Damage

RUNX2 has been considered to be one of the master regulators for osteoblast differentiation and essential for bone formation as well as mineralization *in vivo* [41, 119]. In accordance with this notion, RUNX2 has an ability to transactivate a number of osteogenic markers such as *type 1 collagen*, *osteopontin*, and *osteocalcin* [120]. In a sharp contrast to RUNX1 and RUNX3, oncogenic property of RUNX2 has been postulated in several human cancers. It has been shown that *RUNX2* gene amplification is frequently observed in human osteosarcoma [121]. Additionally, accumulating evidence demonstrated that the dysregulation of *RUNX2* expression is frequently detectable in a variety of human cancers and higher expression level of *RUNX2* is closely correlated with poor clinical outcome of the patients [122–124]. However, the precise molecular mechanism(s) how RUNX2 could contribute to the initiation and progression of cancers remains elusive. Of note, Blyth et al. described that RUNX2 provides a strong anti-apoptotic signal even in the presence of functional p53, indicating that RUNX2 neutralizes p53 [125]. Westendorf et al. revealed that RUNX2 represses p53-target gene *p21* ^*WAF1*^ expression [126]. Thus, it is likely that there is a functional interaction between RUNX2 and p53, and we have then investigated whether RUNX2 could participate in the regulation of p53-dependent DNA damage response. 

**Figure 4:**
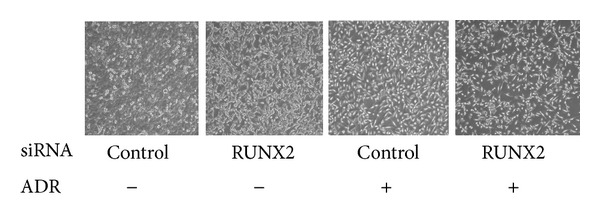
Knockdown of RUNX2 enhances ADR-mediated apoptotic cell death in U2OS cells. U2OS cells were transiently transfected with control siRNA or with siRNA targeting RUNX2. Twenty-four hours after transfection, cells were exposed to ADR or left untreated. Twenty-four hours after ADR exposure, cells were observed by phase-contrast microscopy.

 Under our experimental conditions, we have found the complex formation between p53 and RUNX2 in ADR-treated U2OS cells but not in untreated cells. RUNX2/p53 complex was recruited onto the various p53-responsive promoters including *p21* ^*WAF1*^ and *BAX* in response to ADR. Forced expression of RUNX2 downregulated the transcription of p53-inducible genes, and RUNX2 knockdown further elevated the expression levels of p53-target genes in response to ADR as compared with those in ADR-treated cells expressing the endogenous RUNX2. Moreover, knockdown of RUNX2 significantly enhanced ADR-mediated apoptotic cell death in U2OS cells, whereas RUNX2 knockdown had a negligible effect on *p53*-deficient H1299 cells (Figure 4). Unlike RUNX1 and RUNX3, RUNX2 showed an undetectable effect on ADR-mediated phosphorylation as well as acetylation status of p53. Intriguingly, we have found that RUNX2/p53 complex contains HDAC6 (histone deacetylase 6), and HDAC6-specific chemical inhibitor termed tubacin treatment enhances ADR-mediated induction of p53-responsive gene expression, indicating that deacetylase activity of HDAC6 is required for RUNX2-mediated downregulation of p53-target genes. Taken together, our results implicate that, unlike RUNX1 and RUNX3, RUNX2 acts as a negative regulator for p53 in response to DNA damage and might be an attractive novel molecular target for improved therapeutic outcome [127]. 

## 10. Future Perspective

**Figure 5:**
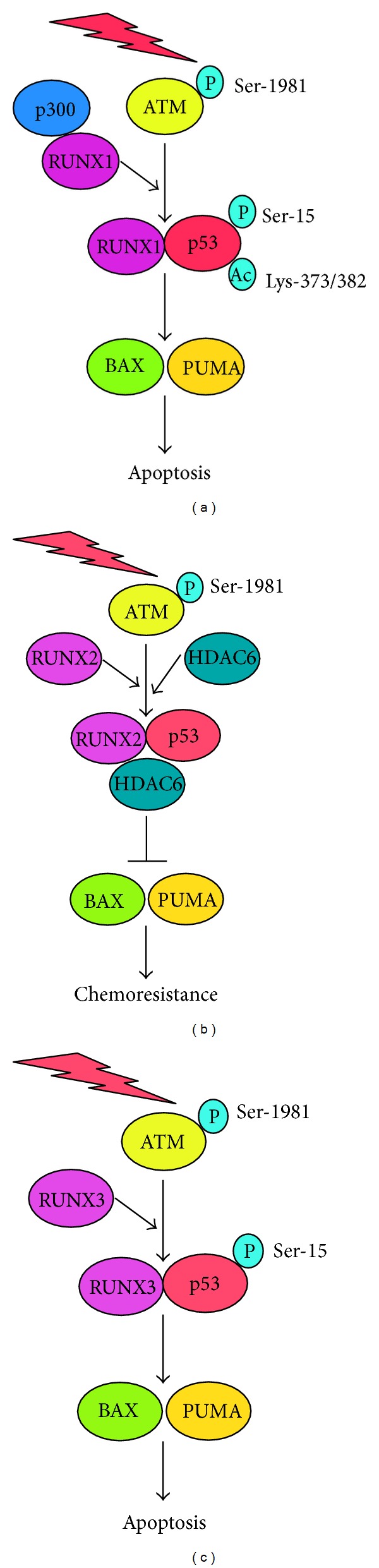
Distinct regulatory role of RUNX family on p53 in response to DNA damage. Under our experimental conditions, RUNX1 as well as RUNX3 acts as a positive regulator for p53 in response to DNA damage, whereas DNA damage-induced proapoptotic activity of p53 is strongly abrogated by RUNX2.

 Maintenance of genomic integrity by the proper DNA damage response is critical to prevent tumorigenesis. Tumor suppressor p53 is the most studied key player which monitors and checks the genomic integrity during DNA damage response and eliminates cells with seriously damaged DNA through apoptotic cell death. RUNX family members are the novel players, which participate in the regulation of p53-dependent DNA damage response. RUNX1 and RUNX3 enhance proapoptotic activity of p53, whereas RUNX2 prohibits p53-dependent apoptotic cell death following DNA damage (Figure 5). Among them, RUNX2 might be an attractive molecular target to improve the conventional chemotherapy. Since siRNA-mediated knockdown of RUNX2 significantly enhanced the antitumor effect of ADR through the activation of wild-type p53, it is likely that the constitutive silencing of RUNX2 expression and/or the inhibition of the transcriptional activity of RUNX2 might be a promising strategy to enhance the antitumor effect of the conventional chemotherapy. To our surprise, knockdown of RUNX2 in certain cancerous cells carrying *p53* mutation further promoted apoptotic cell death in the presence of well-known antitumor drugs (unpublished observations). Therefore, we postulate that the silencing of RUNX2 might be effective on the conventional chemotherapy of malignant tumors regardless of their *p53* status.
